# Acute Systemic Viral Infection Masquerading as an Infiltrating Lymphoma in an Elderly Patient: A Case Report and Review of the Literature

**DOI:** 10.1155/2013/318358

**Published:** 2013-02-12

**Authors:** Hani M. Babiker, Troy Wiedenbeck, Ryan S. Robetorye, Utkarsh Acharya, Susan Wilansky, Shimon Kusne

**Affiliations:** ^1^Division of Hematology and Oncology, The University of Arizona Cancer Center, 1515 N Campbell Avenue, Tucson, AZ 85724, USA; ^2^Division of Hospital Medicine, Mayo Clinic, 5777 E Mayo Boulevard, Phoenix, AZ 85054, USA; ^3^Division of Cardiology, Mayo Clinic, 5777 E Mayo Boulevard, Phoenix, AZ 85054, USA; ^4^Department of Laboratory Medicine and Pathology, Mayo Clinic, 5777 E Mayo Boulevard, Phoenix, AZ 85054, USA; ^5^Division of Infectious Diseases, Mayo Clinic, 5777 E Mayo Boulevard, Phoenix, AZ 85054, USA

## Abstract

Primary Epstein-Barr virus (EBV) infection occurs mainly in adolescents and young adults, with more than 90% of adults having serological evidence of past infection. Primary infection in those over the age of 40 is associated with an atypical and often more severe presentation that can lead to more extensive and invasive, and often unnecessary, diagnostic testing. The incidence of severe EBV-related illness in older adults has been observed to be increasing in industrialized nations. The characteristic presentation of infectious mononucleosis (IM) syndrome in elderly patients (age > 65) is not clearly defined in the literature. Here, we describe a case of primary EBV infection in an 80-year-old female and review the literature regarding primary seroconversion in elderly patients.

## 1. Case Report

**Figure 1:**
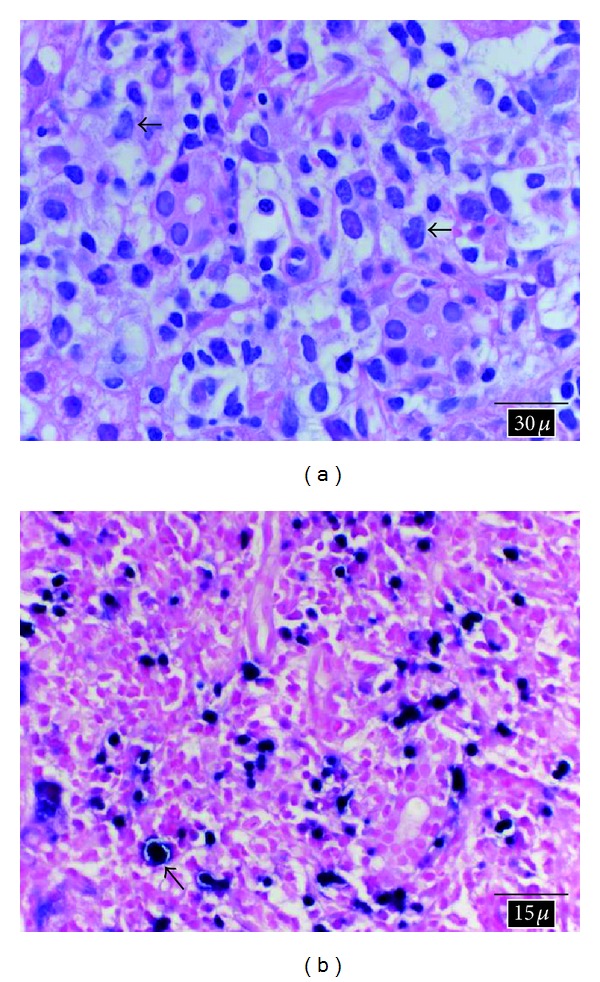
Liver biopsy: (a) high power view of H&E-stained section of liver core biopsy showing focally prominent mixed inflammatory cell infiltrate comprised of small lymphocytes, plasma cells, neutrophils, and rare large cells with irregular nuclear contours and prominent eosinophilic nucleoli. Arrows point to large atypical lymphocytes (100x magnification). (b) EBV in situ hybridization analysis for the detection of EBV-encoded RNA (EBER) reveals abundant positive cells with dark nuclear staining, consistent with EBV infection. Arrow points to a large atypical EBV-positive cell (50x magnification).

**Figure 2:**
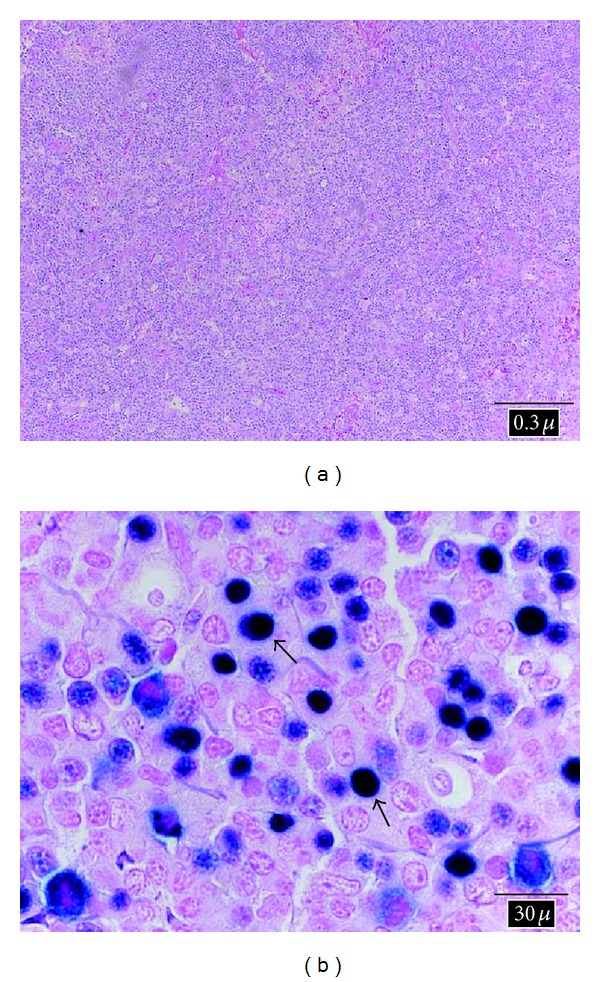
Lymph node biopsy: (a) low power view of H&E-stained section of lymph node biopsy showing diffuse effacement of the normal lymph node architecture by a mixed infiltrate comprised of small lymphocytes, plasma cells, histiocytes, and scattered large cells with vesicular chromatin, irregular nuclear contours, and conspicuous nucleoli (10x magnification). Relatively frequent mitoses were also observed scattered throughout the specimen. (b) EBV in situ hybridization analysis for the detection of EBV-encoded RNA (EBER) reveals abundant positive cells with dark nuclear staining, consistent with EBV infection. Arrows point to large atypical EBV-positive cells (100x magnification).

An 80-year-old female with a history of idiopathic thrombocytopenic purpura and splenectomy presented to our institution with seven days of fevers at night, chills, night sweats, and weight loss. She lived with her husband and was independent in her activities of daily living. She was asymptomatic one week prior to initial presentation to the emergency room. Upon admission, she appeared fatigued with an exam remarkable for icterus, enlarged right neck and left supraclavicular lymph nodes (LNs), hepatomegaly, and right upper quadrant tenderness. Laboratory tests revealed a WBC of 19.7 × 10^9^/L with a differential of 31% neutrophils, 58% lymphocytes, and 11% monocytes. Liver function tests (LFTs) revealed an alkaline phosphatase of 595 *μ*/L, aspartate aminotransferase of 281 *μ*/L, alanine aminotransferase of 200 *μ*/L, and a total bilirubin of 2 mg/dL. Abdominal ultrasound noted multiple enlarged porta hepatis LNs and findings suggestive of choledocholithiasis. MRCP revealed no choledocholithiasis or ductal dilatation but revealed significant interaortocaval, periportal, and common hepatic enlarged LNs. A CT scan of the chest revealed enlarged bilateral axillary and significant anterior and superior mediastinal LNs suggestive of lymphoma. An infectious disease workup was negative for viral hepatitis, mononucleosis (monospot test), coccidiomycosis, ehrlichiosis, rickettsia, QuantiFERON-TB Gold In-Tube, histoplasmosis, blastomycosis, CMV, and toxoplasmosis, and blood cultures were also negative. An excisional biopsy of a right neck LN revealed an atypical lymphoid proliferation. The surgical team initially recommended a mediastinoscopy with mediastinal LN sampling for a more adequate specimen, but a multidisciplinary group meeting concluded that ultrasound-guided needle percutaneous liver parenchyma and periportal LNs biopsies should be performed due to lower invasiveness and higher diagnostic yield potential. Liver and retroperitoneal LN core biopsies revealed an atypical lymphoid infiltrate (Figures 1(a) and 2(a)). EBV anti-EBNA and anti-VCA IgG/IgM were nonreactive; however, serum EBV DNA by PCR was elevated (146,500 copies/mL), suggesting acute systemic EBV infection. Furthermore, in situ hybridization analysis of the liver and LN tissue specimens was diffusely positive for EBV-encoded RNA (Figures 1(b) and 2(b)), further confirming EBV as the cause of her constellation of symptoms in our seronegative patient. Immunoperoxidase stains of tissue specimen revealed an atypical lymphoid infiltrate composed of a mixed population of both B- and T-cells. A Ki-67 stain indicated that the infiltrate appeared proliferative. Flow cytometric immunophenotyping identified lymphocytes comprising 78% of total cells and consisted of a mixture of phenotypically unremarkable T-cells (66%), polytypic B-cells (32%), and NK cells (2%). There did not appear to be atypical expression of CD4 and CD8 stains. The histologic and flow cytometric immunophenotyping were not diagnostic of a B-cell, T-cell, or Hodgkin's lymphoma. The patient was started on ganciclovir and subsequently switched to valganciclovir for 3 weeks on discharge. At the time of discharge (after a week of hospitalization) and during followup, the patient was asymptomatic and showed improvement in her liver function test (LFT). 

## 2. Discussion

EBV is the cause of IM and is a virus of the family Herpesviridae, which consists of a linear DNA core surrounded by a nucleocapsid and an envelope that contains glycoprotein. IM occurs most commonly in younger children, with a second peak in adolescence. By adulthood, 90% of individuals have been infected and have antibody to the virus. The incidence of IM in persons over the age of 35 is two to four per 100,000 per year [1]. Young children are asymptomatic or present with mild pharyngitis, fever, fatigue, and myalgia. In contrast, elderly patients present with longer fevers, jaundice, lymphadenopathy, and hepatomegaly [1, 2]. A study of 27 patients with IM aged 40 to 72 years revealed fever during the entire illness occurring in about 93% of elderly patients, with 37% of the patients exhibiting fevers for 14 days [3]. Approximately 52% of the patients in the study also required hospitalization. Posterior cervical, anterior cervical, and absence of lymphadenopathy occurred in 40%, 16%, and 44% of elderly patients, respectively. Elderly patients with IM also have a higher prevalence of hyperbilirubinemia, with levels greater than 2 mg/dL occurring in 30% of patients relative to 3% in patients 35 years of age and younger [1]. The literature review of case reports of elderly patients with IM also revealed significant elevations in bilirubin and LFTs (Table 1). These findings often erroneously mislead clinicians toward a diagnosis of choledocholithiasis [3–5]. Elderly patients with IM consistently lack lymphocytosis, and the prevalence of heterophile-negative IM increases with age [1, 6–8]. A study of elderly patients with IM revealed positive titers of anti-EBV-VCA IgM, anti-EBV-VCA IgG, anti-EBV EA-IgG, and anti-EBV-EBNA in 55.6%, 88.9%, 85.2%, and 92.6% of patients, respectively [3]. In contrast to all previously published cases, our patient had negative serologies but showed significant elevation in EBV viral load. Conclusively, in comparison to younger patients with IM elderly patients tend to present with longer duration of illness with prolonged fevers, jaundice, hepatosplenomegaly, generalized lymphadenopathy, and rashes. Laboratory results frequently reveal marked hyperbilirubinemia, significant elevation in LFT, negative heterophile-antibody test, and lack of lymphocytosis (Table 2).

EBV infection is associated with the development of B-cell and T-cell non-Hodgkin lymphoma, Hodgkin lymphoma, nasopharyngeal carcinoma, and posttransplant lymphoproliferative disorders [9, 10]. Subsets of patients also develop chronic-active EBV infection that involves progressive and recurrent IM symptoms with organ involvement and high EBV viral load over a longer period [11]. Treatment of EBV IM is usually supportive, and most cases of hepatitis resolve spontaneously. The use of steroids in acute EBV infection is controversial and should be considered only in patients with life-threatening infections. Antiviral medications and steroids reduce oropharyngeal shedding of the virus temporarily but have no clinical benefit compared to placebo [12]. Our patient was successfully treated with antiviral therapy, which resulted in prompt resolution of her presenting complaints. The literature review revealed that supportive care was the mainstay of treatment in elderly patients with EBV IM (Table 1). Presumptive diagnoses such as lymphoma secondary to significant lymphadenopathy, leukemia due to presence of atypical lymphocytosis, choledocholithiasis, or fever of unknown origin (FUO) can lead to delayed diagnosis and unnecessary invasive procedures. These unnecessary procedures in elderly patients with IM are due to both older age at presentation and atypical clinical presentation. Performed procedures include excisional biopsies, bone marrow aspiration and biopsies, bronchoscopies, and endoscopic retrograde cholangiopancreatography [1, 3]. Consideration of this disease entity is essential in elderly patients with presumptive diagnoses of lymphoma, chronic lymphocytic leukemias, cholestatic jaundice, and FUO. Tests for specific EBV antibodies should be obtained, and if negative and clinical suspicion remains high, EBV viral load PCR analysis should be performed. Our case highlights the benefit of antiviral treatment for severe disease in immunocompromised elderly patients, as our patient had a recovery in one week. In addition, our study highlights the importance of a multidisciplinary approach when caring for elderly patients with unusual clinical presentations.

## Figures and Tables

**Table 1:** The literature review of case reports of elderly patients (>65) with primary EBV infectious mononucleosis.

Age	Gender	Presenting symptoms and signs	AST/ALT (units/L)	Tbili (mg/dL)	ALKP/GGT (units/L)	Atypical lymphocytes	Serology/PCR	Radiographic studies	Liver biopsy results	Treatment and outcome
68	M	Malaise and jaundice.	1553/1396	Tbili 34 Dbili 25	147/50	Present	Pos anti-VCA IgM and negative anti-EBNA.	Normal imaging.	No biopsy performed.	Supportive management. Improved during hospitalization [13].

66	F	Malaise, anorexia, and jaundice.	3090/1490	Tbili 26.4 Dbili 15.1	318/no GGT	Present	Pos monospot test, anti-VCA IgM/IgG, and EBV PCR.	Abd US chronic liver disease changes.	Hepatitis and cirrhosis.	Prednisolone for AIH. Asymptomatic at 2-month followup [14].

70	F	Fever, pharyngitis, and back pain.	339/390	Dbili 0.4	524/no GGT	Present	Pos heterophile antibody and anti-VCA IgM/IgG.	No radiographic studies.	No Biopsy performed.	Supportive treatment [1].

72	F	Sore throat, fever jaundice, splenomegaly, and fever.	904/1258	Tbili 21.4 DBili 15.9	370/250	Absent	Pos anti-VCA IgM, EBV PCR, anti-EBEA IgM. Neg anti-VCA IgG.	Normal imaging.	No Biopsy performed.	Supportive management. Fatigue at 6-month followup [4].

67	F	Sore throat, fever, jaundice, and RUQ pain.	847/499	Tbili 11.4 Dbili 8.6	1389/198	Present	Pos monospot test and anti-VCA IgM/IgG.	Normal imaging.	Lymphocytic infiltration in portal tracts.	Supportive management. Asymptomatic at 3-month followup [4].

83	M	Night sweat and weight loss.	337/407	Tbili 10.5	512/no GGT	Present	Pos heterophile antibody, anti-VCA IgM/IgG, and EBV PCR.	CT revealed splenomegaly and lymphadenopathy.	No biopsy performed.	Supportive management. Asymptomatic at 1 month [15].

72	M	Loss of appetite and jaundice.	ALT-254	Tbili 2.9	677/817	Present	Pos anti-VCA IgM/IgG and anti-EBNA IgG.	CT revealed splenomegaly.	No biopsy performed.	Supportive management. Asymptomatic at 4 months [16].

76	F	Falls and dizziness.	Not reported	Tbili 5.7 Dbili 1.5	Not reported	Absent	Pos anti-VCA IgM/IgG. Neg anti-EBEA and anti-EBNA.	CT revealed aortic lymphadenopathy.	No biopsy performed.	Supportive management. Asymptomatic at 6 months [17].

73	F	Falls and dizziness.	Not reported	Tbili 1.2 Dbili 0.3	Not reported	Absent	Pos anti-VCA IgM/IgG. Neg anti-EBEA and anti-EBNA.	Normal imaging.	No biopsy performed.	Supportive management. Asymptomatic at 6 months [17].

Abd US: abdominal ultrasound; AIH: autoimmune hepatitis; ALKP: alkaline phosphatase; ALT: alanine aminotransferase; anti-EBEA: anti-EBV early antigen antibody; anti-EBNA: anti-EBV-associated nuclear antigen antibody; anti-VCA: EBV anti-viral caspid antigen antibody; AST: aspartate aminotransferase; Dbili: direct bilirubin; GGT: gamma glutamyl transpeptidase; HIV: human immunodeficiency virus; IgG: immunoglobulin G; IgM: immunoglobulin M; Neg: negative; PCR: polymerase chain reaction; Pos: positive; Tbili: total bilirubin.

References: Dogan et al., 2007 [13], Koay et al., 2008 [14], Axelrod and Finestone, 1990 [1], Tahan et al., 2001 [4], Malfuson et al., 2011 [15], Thoufeeg et al., 2007 [16], and Dourakis et al., 2006 [17].

**Table 2:** Common characteristics of infectious mononucleosis in elderly patients in comparison to younger patients.

Symptoms and signs
Jaundice
Prolonged fevers
Hepatosplenomegaly
Generalized lymphadenopathy
Rashes
Rarely neurological symptoms
Laboratory tests
Lack of lymphocytosis
Heterophile-negative antibody test (increases with age)
Significant hyperbilirubinemia
Elevation in liver function tests
Positive EBV serology
Outcome
Protracted duration of illness
Prolonged hospitalizations
Common workup
Blood tests including serologies
Lymph nodes/liver biopsies
Bone marrow aspiration and biopsies
Imaging studies including MRI/CT scans
Bronchoscopies
ERCP

ERCP: endoscopic retrograde cholangiopancreatography. References: Horwitz et al., 1983 [3], and Axelrod and Finestone, 1990 [1].
